# Physical activity during pregnancy and association with changes in fat mass and adipokines in women of normal-weight or with obesity

**DOI:** 10.1038/s41598-021-91980-z

**Published:** 2021-06-15

**Authors:** Ulrika Andersson-Hall, Hanna de Maré, Freja Askeli, Mats Börjesson, Agneta Holmäng

**Affiliations:** 1 Department of Physiology, Institute of Neuroscience and Physiology, Sahlgrenska Academy, University of Gothenburg, Box 432, 405 30 Gothenburg, Sweden; 2 Department of Food and Nutrition, and Sport Science, Centre for Health and Performance, University of Gothenburg, Gothenburg, Sweden; 3 Department of Acute and Molecular Medicine, Institute of Medicine, Sahlgrenska Academy, University of Gothenburg, Gothenburg, Sweden; 4 Department of MGA, Sahlgrenska University Hospital, Region of Västra Götaland, Gothenburg, Sweden

**Keywords:** Physiology, Endocrinology

## Abstract

Adipose tissue and adipokine concentrations change markedly during pregnancy, but the effects of physical activity on these changes are rarely studied. We aimed to assess physical activity levels in pregnant women of normal-weight (NW) or with obesity (OB), and to determine the relation with changes in fat mass and adipokines. In each trimester, pregnant women (136 NW, 51 OB) were interviewed about their physical activity and had their body composition, leptin, soluble leptin receptor (sOB-R) and adiponectin determined. NW reported higher activity and more aerobic exercise than OB during early pregnancy. Both groups maintained training frequency but reduced overall activity as pregnancy progressed. NW women reporting aerobic and/or resistance exercise and OB women reporting aerobic exercise had greater sOB-R increases (independent of BMI or gestational weight gain). In NW, exercise also associated with lower fat mass and leptin increases. Higher activity levels associated with lower gestational weight gain in both groups. The relationship between physical activity and adiponectin differed between NW and OB. Maternal exercise may partly mediate its beneficial effects through regulation of leptin bioavailability, by enhancing pregnancy-induced increases in sOB-R. This could be of particular importance in OB with pre-gestational hyperleptinemia and leptin resistance.

## Introduction

Both a high pre-gestational BMI and excess gestational weight gain affect maternal as well as fetal health outcomes^1,2^. Maternal risks include gestational diabetes, hypertension/pre-eclampsia, adverse obstetric outcomes, and post-pregnancy weight retention, whereas the fetus is at higher risk of being born large-for-gestational age (LGA) and of later life obesity, metabolic syndrome and diabetes. Even though pregnant women receive guidelines for optimal gestational weight gain in combination with dietary and physical activity advice, a large proportion of women gain more weight than the guidelines set by the Institute of Medicine (IOM)^3^.

Exercise recommendations have changed dramatically in the past few decades, from the 1950s when maternal exercise was considered dangerous and should be discouraged to recent guidelines emphasizing beneficial effects of maternal exercise and harmful effects of a sedentary lifestyle on maternal and fetal health^4^. Current guidelines recommend a minimum of 150 min moderate intensity exercise per week and 2–3 resistance training sessions per week^5,6^.

Maternal exercise may limit excessive weight gain but can also have positive metabolic effects on mother and fetus independent of weight changes^7–10^. Exercise has been shown to decrease the risk for maternal conditions such as pre-eclampsia and gestational diabetes, especially in women with overweight or obesity^8^, and has been shown to increase placental function^11,12^. Furthermore, exercise during pregnancy lowers the risk of children being born LGA and decreases neonatal fat mass^13^. Numerous rodent studies have also shown positive long term effects of maternal exercise on offspring metabolic health such as prevention of obesity and diabetes^14^, with protection lasting up to three generations after maternal exercise was imposed^15^. The mechanisms by which exercise stimuli affect developmental programming are not fully established, but the placental-foetal system is thought to play a central role^14^.

Pregnancy is associated with a range of metabolic and endocrine adaptations that allow women to meet their own energy needs and those of the growing fetus. These changes include increased fat mass and changes in circulating adipokines^16^. Leptin concentrations are elevated during pregnancy due to secretion from the placenta, as well as from the increasing stores of adipose tissue^17^. The soluble leptin receptor (sOB-R), which binds to circulating leptin to form leptin-sOB-R complexes, also increases during pregnancy^18–20^. The upregulation of sOB-R is thought to be important for pregnancy associated adaptations of leptin bioavailability and leptin resistance, and thereby for the regulation of maternal energy balance and placental nutrient transport^17^. We have also previously shown that the ratio of leptin to sOB-R predicts insulin resistance during pregnancy and that upregulation of maternal sOB-R associated with lower infant fat mass^20,21^.

We hypothesize that beneficial effects of maternal exercise on maternal and fetal metabolism could partly be mediated through regulation of fat mass and adipokine levels, perhaps predominantly in women with obesity who exhibit excess adiposity and underlying hyperleptinemia and leptin resistance. Some previous studies have shown a leptin lowering effect of maternal exercise, whereas others showed no effect^7,22–25^. There are, however, no studies to date investigating how maternal exercise or physical activity associates with pregnancy related changes in sOB-R, which might be of major regulatory importance for leptin function. Our objective was therefore to study self-reported physical activity and exercise training during pregnancy in women of normal-weight or with obesity, and to determine its relation to changes in fat mass and adipokines.

## Methods

### Ethics

The study was approved by the Regional Ethical Review Board in Gothenburg (Dnr 402–08) and was performed in accordance with relevant guidelines and regulations. All women received oral and written information about the study and signed an informed written consent-form before enrolment.

### Subjects

Pregnant women of normal-weight (NW: BMI 18.5–24.9 kg/m^2^) or with obesity (OB: BMI ≥ 30 kg/m^2^), aged 20–45 years, were recruited from six antenatal health units within the Gothenburg area as part of the Pregnancy Obesity Nutrition and Child Health (PONCH) study, as previously described^20,21^. Exclusion criteria in the original study were non-European descent (due to large differences in body composition and its associated metabolic risk between ethnicities^26^), having any form of diabetes mellitus (type-1-diabetes, type-2-diabetes or gestational diabetes), other chronic diseases or pregnancy related complications, use of tobacco or neuroleptic drugs and vegetarianism or veganism. The women attended three study visits during pregnancy (weeks 8–12 [trimester one, T1], 24–26 [two, T2] and 35–37 [three, T3]) at the Sahlgrenska University Hospital. Data for this study was collected between April 2009 and October 2019. The number of women that attended all three visits and were included in the study were 136 NW and 50 OB.

### Study visits

Visits during pregnancy have been described previously^27^. In brief, all visits took place in the morning after an overnight fast and included anthropometric and body composition measurements, blood sampling, and completion of life-style questionnaires. As part of the study, NW and OB pregnant women were randomized into dietary intervention or control subgroups^27^; the intervention subgroup received dietary counseling to increase adherence to Nordic Nutrition Recommendations. No guidance or recommendations regarding physical activity was given, and there were no differences in study outcomes between control and intervention groups in either the NW or OB group (for original study outcome of body weight or fat mass change, nor for self-reported physical activity levels or number of exercise sessions). The data for control and intervention groups have therefore been pooled in their respective BMI categories.

### Self-reported physical activity and training

At the visit, the women were interviewed about their physical activity level and exercise training. At the T1 visit, the questions related to the last 12 months. At the T2 and T3 visits, the questions related to the time since the last visit (3 months). Firstly, the women were asked to rank their level of physical activity during leisure time and during work using the Saltin-Grimby physical activity level scale (SGPALS), which has been validated in a large number of studies both for reproducibility, for concurrent validity against maximal oxygen uptake and predictive validity for disease risk factors^28^. The physical activity of the SGPALS during leisure time is graded 1–4 (in brief: level 1 = sedentary, 2 = some physical activity, 3 = regular physical activity and exercise, 4 = regular hard physical training). They were also asked what mode of transportation they used in the leisure time and to/from work.

Secondly, for estimation of exercise training, the women were asked how often they trained (times/week), how long they trained (min/session) and which type of exercise they performed. The type of exercise reported were at the time of analysis organized into 3 categories; “walk”- walking or low impact (mainly walking indoor/outdoor and a few women performing pregnancy water movement classes), “aerobic” (aerobic exercises such as tennis, jogging, treadmill, swimming, biking, football, and fitness classes focusing on aerobic exercise), and “resistance” (strength training such as weight lifting or bodyweight resistance training, but also general fitness classes with large focus on resistance training).

### Body composition and biochemical measurements

Body composition was measured by air-displacement plethysmography using the Bod Pod Gold Standard system (Bod Pod 2007 A, Life Measurement, Concord, CA, software versions 4.2.0 and 5.2.0.) using gestational-age specific equations according to our previously described protocol^29^. Leptin and adiponectin were analysed at the Clinical Chemistry Laboratory, Sahlgrenska University Hospital (accredited in accordance with the International Standard ISO 15189:2007). Leptin (Human Leptin Quantikine, R&D Systems, Minneapolis, MN; interassay coefficient of variation, 8.0% at 9 μg/l), the soluble leptin receptor (sOB-R) (Human Leptin R Quantikine, R&D Systems) and adiponectin (Human Adiponectin ELISA kit, Millipore, Billerica, MA; interassay coefficient of variation, 7.0% at 10.5 mg/l) were analysed using ELISA. sOB-R measurements were conducted for a subset of women, i.e. the women that were included in the full PONCH study of both mother and child^21^; this resulted in 84 NW women and 33 OB women that had complete sOB-R measurements in both T1 and T3.

### Statistical analysis

Categorical variables were expressed as number and percentage, and continuous variables as mean ± standard deviation. Differences between NW and OB groups with respect to ordered categorical variables were assessed using the chi-square test or Fisher´s exact test; between-group differences for continuous variables were assessed using t-test. Within-group comparisons of activity levels and number of training sessions over time were assessed using Friedman’s test for ordinal numbers (and Wilcoxon tests for post hoc analysis). Differences in metabolic changes depending on activity level or whether women performed a training modality were determined using ANCOVA adjusted for maternal age, BMI, parity and educational level. For baseline associations between training amount and metabolic variables in T1, Spearman correlations were used (number of training sessions was not normally distributed). All tests were two-tailed and conducted at the 0.05 significance level.

For sOB-R, a subset of women was analyzed as described above. Failure analysis for women that were included for sOB-R analysis compared with women that were not showed no difference in BMI, fat mass, leptin or adiponectin, either for T1-values or for changes T1-T3 (Supplemental table S1).

## Results

### Maternal characteristics

The NW and OB groups did not differ in age or parity, but educational level was higher in the NW group compared with the OB group (Table 1). The NW group had lower BMI, fat mass and leptin level, but higher sOB-R and adiponectin levels. NW had a higher gestational weight gain than OB, but compared to IOM recommendations had fewer women gaining excessively.

**Table 1:** Maternal characteristics.

	NW n = 136	OB n = 50	*p*
	Mean ± SD	Mean ± SD	
Age (years)	31.0 ± 3.6	31.1 ± 3.4	0.784
Parity (0/1/2/3) %	54/36/10/0	52/38/6/4	0.109
Educational level (2/3/4) %	11/15/74	32/18/50	0.003
BMI (kg/m^2^)	22.1 ± 1.5	34.4 ± 3.9	< 0.001
GWG (kg)	11.4 ± 2.9	7.8 ± 4.3	< 0.001
GWG > IOM guidelines %	8	42	< 0.001
**Trimester 1 measurements**			
FM (kg)	16.7 ± 4.1	46.6 ± 10.4	< 0.001
Leptin (ng/ml)	13.0 ± 6.5	50.3 ± 17.5	< 0.001
sOB-R (ng/ml)*	42.6 ± 13.2	23.2 ± 5.6	< 0.001
Adiponectin (ug/ml)	17.7 ± 7.4	11.1 ± 5.6	< 0.001

*p* denotes significance between NW and OB using t-test for continuous variables, Chi square test for parity and education, and Fisher’s exact test for GWG > IOM guidelines. Educational levels: 2 = graduated from 3 year Swedish gymnasium (equivalent to upper secondary school), 3 = less than 3 years of university, 4 = more than 3 years of university. *sOB-R analysis was made on a subset of women; n = 84 for NW. GWG, gestational weight gain (from trimester 1 to 3), IOM, Institute of Medicine; NW, women of normal weight; OB, women with obesity.

### Physical activity and exercise training in NW and OB groups

**Figure 1:**
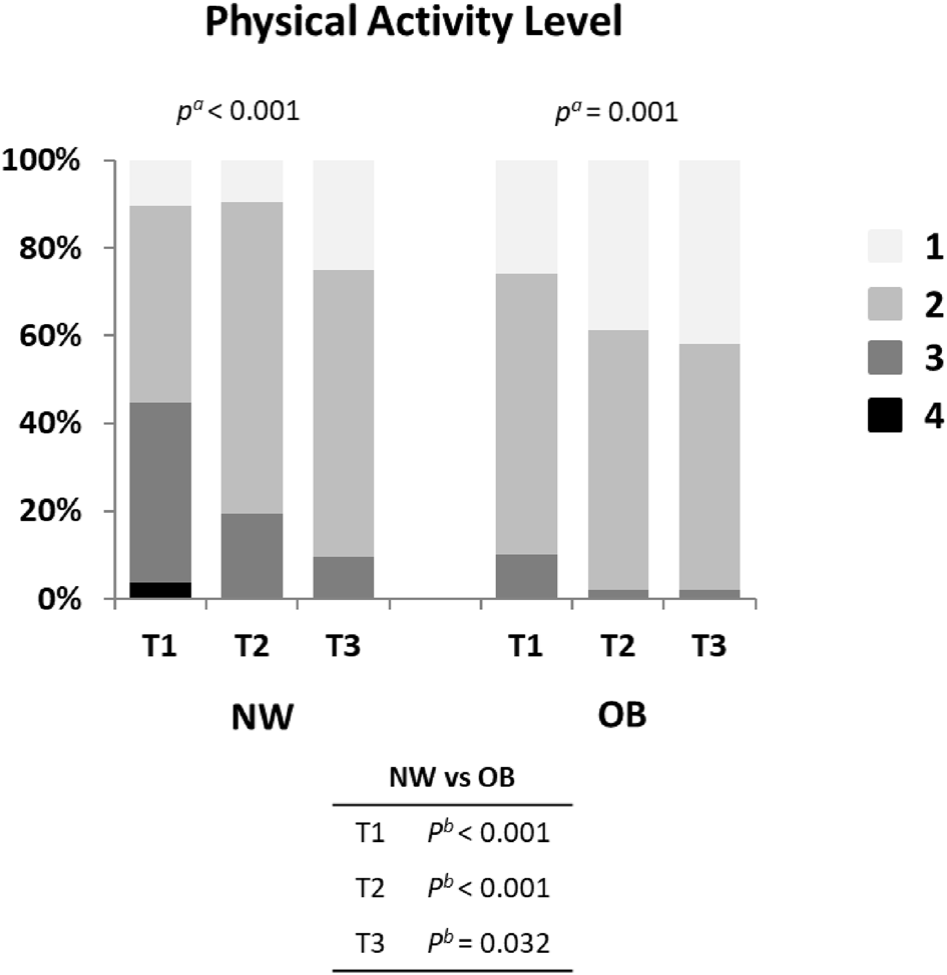
Physical activity levels during pregnancy. The women were asked to rank their level of physical activity during leisure time using the Saltin-Grimby physical activity level scale (in short: level 1 = sedentary, 2 = some physical activity, 3 = regular physical activity and exercise, 4 = regular hard physical training). NW, women of normal-weight; OB, women with obesity; T1, trimester 1; T2, trimester 2; T3, trimester 3. ^a^ Significance using Friedmans test over trimesters 1–3. ^b^ Significance between NW and OB using Chi^2^ tests.

Self-reported activity levels during leisure time were higher in NW compared with OB in all trimesters and decreased significantly over time from T1 to T3 in both NW and OB (Fig. 1).

**Figure 2:**
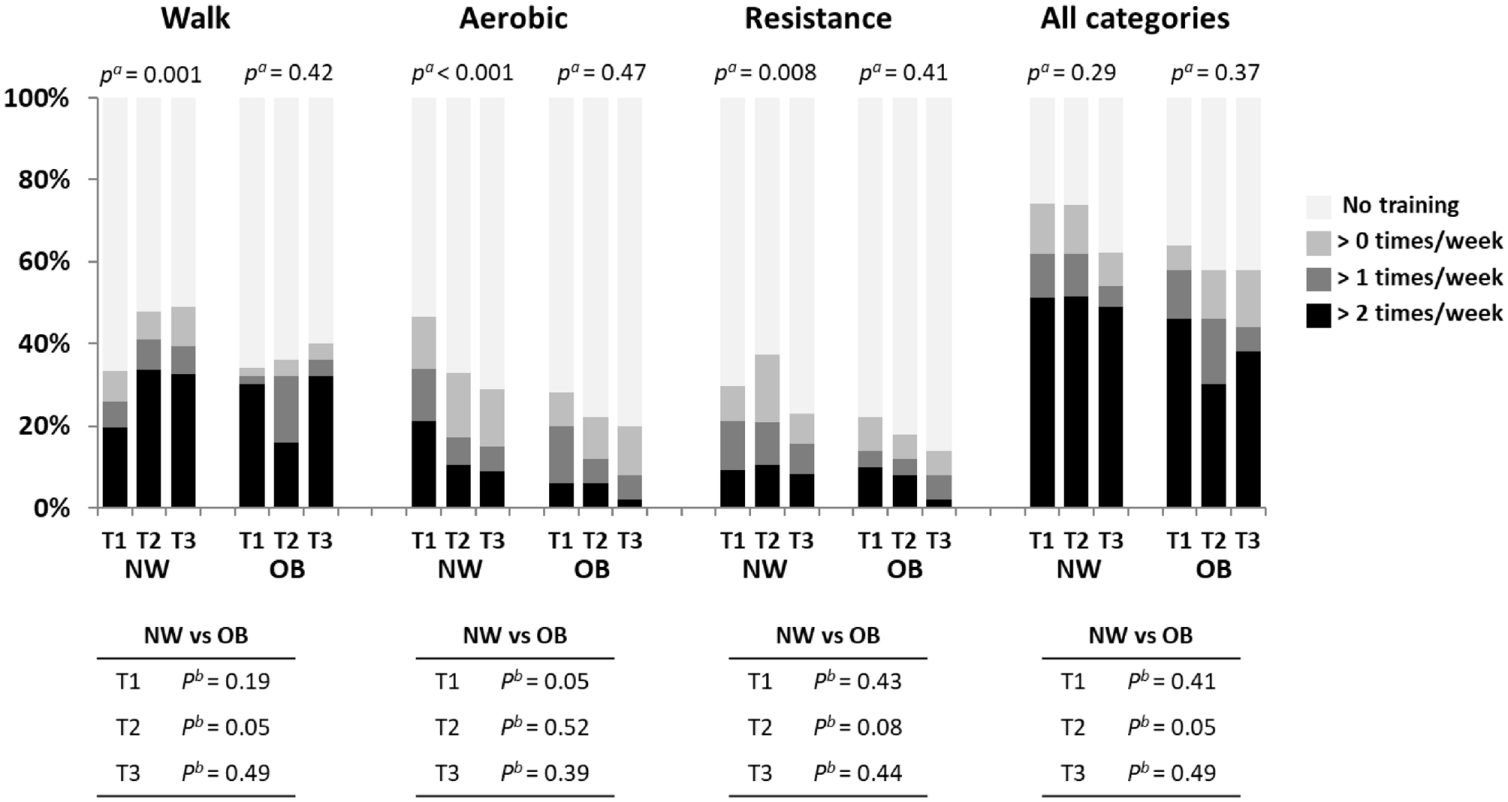
The number of training sessions per week during pregnancy in women of normal-weight or with obesity. The self-reported exercise was categorized into “walk”, aerobic” or “resistance” type training. ^a^Significance using Friedmans test over trimesters 1–3. ^b^Significance between NW and OB using Chi^2^ tests. NW, women of normal-weight; OB, women with obesity; T1, trimester 1; T2, trimester 2; T3, trimester 3.

When reporting exercise training, the total number of training sessions were higher in NW compared with OB in trimester 2 (Fig. 2). When divided into different training modalities, there were no significant differences between NW and OB in resistance sessions, but a higher number of aerobic sessions in T1 and of walk sessions in T2 in NW compared with OB.

For NW, even though there was no change in number of total training sessions over the course of pregnancy, the women increased the number of walk sessions and decreased the number of aerobic sessions from T1 to T3. Resistance training was highest in T2. For OB, there were no differences between trimesters in any training categories.

When analyzing how many women had reached 150 min of total reported exercise per week, we found that more NW than OB women reported at least 150 min in trimester 2 (Table 2). On removing the walk category and reanalyzing the number of women that reached at least 150 min of aerobic and/or resistance training per week, NW were more active in T1 compared with OB. Here, we also saw a significant reduction between trimesters for the NW group. On analyzing how many women were sedentary (no exercise training and no walking or cycling), fewer NW women were inactive compared with the OB group in trimesters 2 and 3.

**Table 2:** Number of women reaching 150 min of exercise and number of women being sedentary.

	NW	OB	*p*
	n (%)	n (%)	
** ≥ 150 min training—all categories**			
Trimester 1	52 (39.4)	16 (32.0)	0.395
Trimester 2	60 (44.8)	13 (26.0)	0.027
Trimester 3	56 (41.5)	14 (28.0)	0.124
** ≥ 150 min training—minus walking**			
Trimester 1	34 (25.0)	5 (10.0)	0.026
Trimester 2	24 (17.8)*	4 (8.0)	0.111
Trimester 3	16 (11.8)**	2 (4.0)	0.162
**Sedentary**			
Trimester 1	6 (4.4)	6 (12.0)	0.088
Trimester 2	4 (3.0)	8 (16.0)	0.004
Trimester 3	12 (8.8)	11 (22.0)	0.023

NW, women of normal weight; OB, women with obesity.

*p* denotes significance between NW and OB using Fisher’s exact test.

*Significance vs trimester 1 using Friedman test (Wilcoxon signed rank test post hoc).

**Significance vs trimester 1 and 2 using Friedman test (Wilcoxon signed rank test post hoc).

### Relationship between physical activity and metabolic measurements

#### Associations at the start of pregnancy

The relationship between starting metabolic parameters measured at T1 and physical activity reported at T1 (for the prior 12 months) are shown in supplemental Table S2. There were no significant associations for NW. For OB women, both the number of aerobic and the number of resistance type training sessions correlated with higher sOB-R.

#### Metabolic changes during pregnancy by activity level

Changes in metabolic measurements from T1 to T3 depending on self-reported leisure time activity are described in Table 3. For both NW and OB women, the gain in body weight was highest in the low activity group according to activity levels reported at the T2 visit (for the period between T1 and T2 visits). For NW women, the body weight gain was also dependent on activity level reported later in pregnancy at T3. Furthermore, in NW women, the soluble leptin receptor sOB-R increased more in women reporting higher activity levels both early and late in pregnancy. Adiponectin, however, decreased more in the higher activity groups.

**Table 3:** Gestational changes in metabolic measurements dependent on self-reported activity levels.

	Trimester 2 Questionnaire Physical activity level				Trimester 3 Questionnaire Physical activity level			
	Level 1	Level 2	Level 3–4	*p*	Level 1	Level 2	Level 3–4	*p*
**NW**	n = 13	n = 96	n = 26		n = 34	n = 89	n = 13	
Δ Body weight (kg)	11.5 ± 2.7	11.8 ± 2.8	10.1 ± 2.8	0.008	12.0 ± 2.7	11.4 ± 2.9	9.4 ± 3.2	0.003
Δ Fat mass (kg)	5.0 ± 2.8	4.8 ± 2.6	3.6 ± 3.3	0.135	4.6 ± 2.4	4.8 ± 2.8	2.8 ± 3.6	0.051
Δ Leptin	7 ± 12	8 ± 12	4 ± 6	0.275	8 ± 10	8 ± 12	3 ± 7	0.373
Δ sOB-R*	11 ± 6	10 ± 13	25 ± 17	< 0.001	10 ± 10	11 ± 13	29 ± 21	< 0.001
Δ Adiponectin	3.0 ± 15.5	− 4.5 ± 6.0	− 4.9 ± 5.0	0.002	− 1.1 ± 12	− 4.5 ± 4.9	− 5.2 ± 6.7	0.177
**OB**	n = 19	n = 29	n = 0		n = 20	n = 29	n = 0	
Δ Body weight (kg)	10.3 ± 4.8	7.5 ± 5.0	–	0.038	10.0 ± 5.3	7.5 ± 5.3	–	0.077
Δ Fat Mass (kg)	2.1 ± 3.8	0.4 ± 3.6	–	0.282	1.5 ± 4.2	0.8 ± 3.4	–	0.964
Δ Leptin	5 ± 20	3 ± 13	–	0.495	7 ± 18	2 ± 14	–	0.344
Δ sOB-R*	8 ± 3	11 ± 8	–	0.390	9 ± 7	10 ± 7	–	0.920
Δ adiponectin	− 1.4 ± 3.2	− 0.8 ± 4.8	–	0.768	− 0.4 ± 4.3	− 1.4 ± 4.3	–	0.407

Significance determined with ANCOVA adjusted for age, BMI in trimester 1, parity and education. Δ denotes the gestational change from trimester 1 to trimester 3. *sOB-R analysis were made on a subset of women in, n = 84 for NW (n = 5, 59, 20 for activity levels in trimester 2 and n = 14, 57, 13 in trimester 3) and n = 33 for OB (n = 12, 21 for activity levels in trimester 2 and n = 13, 20 in trimester 3). Physical activity levels were reported according to the Saltin-Grimby physical activity level scale (in short: level 1 = sedentary, 2 = some physical activity, 3 = regular physical activity and exercise, 4 = regular hard physical training). NW, women of normal weight; OB, women with obesity.

#### Metabolic changes during pregnancy depending on exercise training

**Figure 3:**
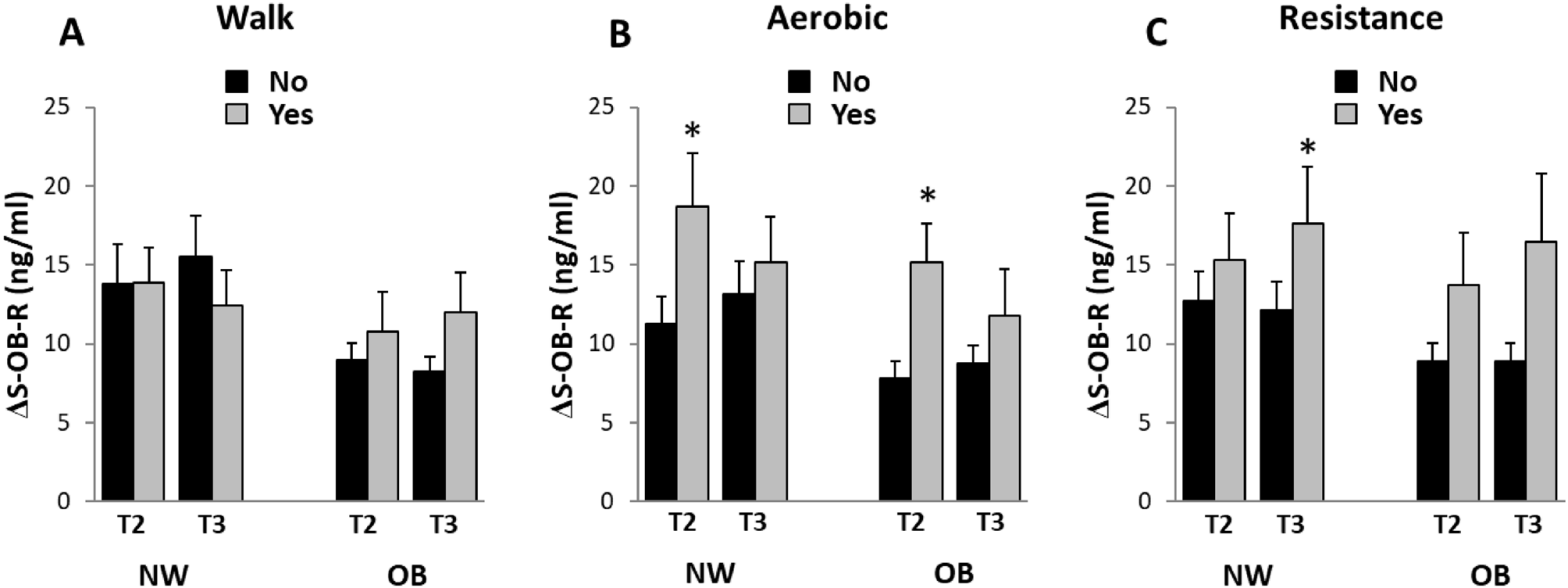
Gestational change in s-OB-R depending on training modality reported during pregnancy. Change in sOB-R (trimester 1 to 3) depending on whether the exercise modality A) “Walk”, B) “Aerobic” or C) Resistance was reported (in trimester 2 or trimester 3). *p < 0.05 using ANCOVA adjusted for age, BMI in trimester 1, parity and education. N = 84 for NW and n = 33 for OB. NW, women of normal-weight; OB, women with obesity; T2, trimester 2, T3, trimester 3.

The metabolic changes were analyzed according to whether or not the women reported a certain type of training. All metabolic changes by reported exercise modality are displayed in supplemental tables S3-S5 with both unadjusted and adjusted p-values. The changes of one metabolic parameter in particular, sOB-R, showed a similar pattern throughout analysis and are presented in greater detail in Fig. 3. Women reporting aerobic training in T2 had a significantly higher sOB-R increase for both NW and OB. Women reporting resistance training in T3 also showed significantly higher increase in sOB-R for NW.

In addition to changes in sOB-R, there were significant differences in weight gain, fat mass gain and change in adiponectin depending on training modality and group (Supplement table S4–S5); For NW, women that reported resistance training in T3 showed a lower gestational gain in weight and fat mass compared to women that did not report resistance training (women reporting resistance training gained 10.5 ± 3.1 kg body weight of which 3.6 ± 3.3 kg was fat mass, women not reporting resistance training gained 11.6 ± 2.8 kg of which 4.9 ± 2.6 kg was fat mass, *p* < 0.05). For OB, women that reported aerobic training in T2 increased their adiponectin levels by 1.7 ± 4.9 ug/ml, whereas women that reported no aerobic training decreased adiponectin by 1.8 ± 3.8 ug/ml (*p* < 0.05).

**Figure 4:**
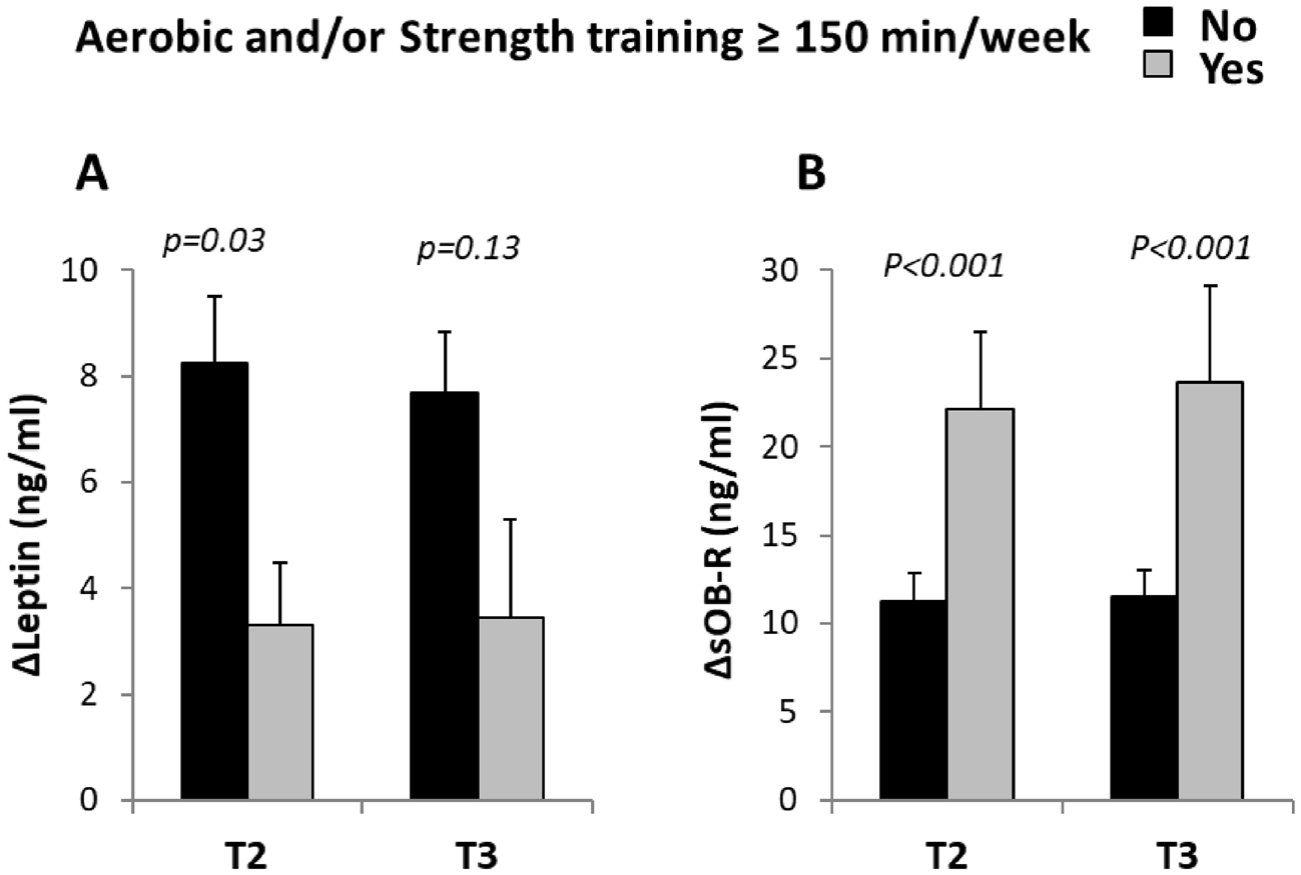
Gestational change in leptin and sOB-R in women of normal weight depending on whether they reached 150 min aerobic and/or resistance training or not. Changes (T1 to T3) in A) leptin and B) sOB-R depending on whether the women reported a minimum of 150 min aerobic and/or resistance training during pregnancy (in T2 and T3). Significance determined with ANCOVA adjusted for age, BMI in trimester 1, parity and education.

The change in metabolic parameters by whether the women reported a minimum of 150 min aerobic and resistance training was analyzed for NW women (the number of OB women reaching 150 min was very small, n = 4, and not analyzed). Women that reported at least 150 min aerobic and/or resistance training in T2 had lower gestational increase in leptin and higher increase in sOB-R compared with women reporting a lower amount of training (Fig. 4). Similarly, reporting at least 150 min training in T3 was also associated with a higher increase in sOB-R. The differences for sOB-R were still significant when adding weight gain as a co-variate (in addition to BMI, age, parity and education), *p* < 0.01 for reporting more than 150 min in T2 and T3.

## Discussion

In this longitudinal pregnancy study, we initially confirmed that women of normal-weight reported a higher leisure-time activity level and more training sessions than women with obesity. As pregnancy progressed, both groups reported decreasing activity levels. Secondly, we investigated the relationship between physical activity and changes in weight, fat mass and adipokines. Higher activity levels were associated with lower gestational weight gain in both groups. Metabolically, high physical activity and exercise training seemed related to leptin regulation. Aerobic training was associated with higher increases in the circulating leptin receptor sOB-R in both groups, and women of normal weight that reported > 150 min of aerobic and/or resistance training showed an exaggerated change in leptin metabolism with both an attenuated leptin increase and a greater sOB-R increase.

In agreement with previous pregnancy studies, we showed that women with obesity were less active, exercised less and were more sedentary compared with women of normal weight^30–33^. Also, in line with previous studies, both groups of women in our study decreased their activity level as pregnancy progressed^34–36^. When we included all types of exercise, the number of women reaching 150 min of exercise per week was 40–45% in the NW group and 26–32% in the OB group depending on trimester. The proportion of women reaching exercise recommendations during pregnancy has varied as widely as 5–38% in previous reports^33,37,38^. The great differences between studies can be explained by different cultures, different time points of pregnancy, slight variations in local recommendations, and importantly by differences in data collection and interpretation methods. The most commonly used guidelines recommend 150 min of moderate aerobic intensity exercise plus strength training^5,6^. In most observational studies so far, the present study included, it is difficult to assess exercise intensity since no objective measurements were used. Walking, for example, often defined as low intensity exercise might in late pregnancy or in a woman with obesity correspond to moderate or high intensity exercise (because of lower relative aerobic capacity). When removing the walking category from accumulated exercise minutes per week, we instead found that only about 11–17% of NW women and 5% of OB women meet the 150 min mark. Similarly, we included strength training in accumulated training time since some of the training forms in the strength category also contained cardiovascular elements. If also removing strength training from the accumulated training time we get an even lower proportion of women reaching 150 min (7–15% for NW and 2–4% for OB, data not shown). Of note, in women of normal weight, we saw a shift from aerobic training to walking the further pregnancy progresses but no change in total number of training sessions. Resistance training was highest in trimester 2, which might be explained by motivational barriers of pregnancy-related illness in trimester 1 and discomfort in trimester 3^39^. In women with obesity, on the other hand, we saw no change in exercise modality between trimesters. These variations in modality through pregnancy suggest that maternal exercise studies should include specification of exercise modality in addition to total training.

Gestational weight gain differed depending on the self-estimated activity level based on the SGPAL Scale^28^. In women of normal-weight, a high reported activity of level 3–4 related to lower weight gain than those with level 1 or 2, whereas for women with obesity an activity level of 2 seemed high enough to reduce weight gain compared with women of level 1 activity. It is important to note that these differences were independent of starting BMI, parity, age or educational level. Our results agree with the combined previous literature, with several reviews and meta-analysis on both observational cohort and interventional studies showing clear associations between physical activity and reduced gestational weight gain^9,40,41^.

The metabolic change that showed most consistent associations with high physical activity was a greater increase in sOB-R, the soluble receptor suggested to mediate leptin bioavailability. In women of normal-weight, we saw a greater sOB-R increase in women who reported higher leisure time activity levels and who performed aerobic or resistance training, irrespective of starting BMI, age, parity or educational level. The upregulation of sOB-R was particularly exaggerated in women that reported at least 150 min of aerobic and/or resistance exercise, where a twofold higher increase was found compared with the women not reaching 150 min. Moreover, in the women training at least 150 min aerobic and/or resistance exercise, there was also a reduced increase in leptin, i.e. a combination of lower leptin and increased receptor results in an even greater reduction of free leptin. We saw similar results in women with obesity, where sOB-R increased most in women reporting aerobic exercise, again in a fully adjusted model. Previous studies examining leptin levels during pregnancy in relation to exercise have either shown no effect of exercise^22–24^ or a leptin lowering effect^7,25^. Our study is the first to investigate the relation between exercise during pregnancy and sOB-R, which in our population seem more sensitive to exercise than leptin itself. A few studies have investigated sOB-R levels in conjunction with exercise in non-pregnant subjects. These showed that exercise increased sOB-R concentration both acutely and over a 5-week intervention^42,43^. In weight loss studies, with intervention by diet and exercise or by bariatric surgery, decreased body weight and fat mass is typically accompanied by increased sOB-R^44^. The situation during pregnancy is different, where increases in sOB-R are paralleled with pregnancy induced fat mass gain^20^. Binding of circulating sOB-R to leptin is thought to regulate the bioavailability of leptin and affects both central and peripheral leptin resistance. This might be particularly important during pregnancy where central leptin resistance ensures adequate maternal food intake and peripheral leptin bioavailability is thought to regulate nutrient transfer across the placenta^17^. Furthermore, high sOB-R levels in pregnancy were shown to have a protective effect on gestational diabetes risk^45^ We have previously shown that sOB-R increases across pregnancy in both our NW and OB cohorts, and that sOB-R upregulation was associated with both an attenuation of pregnancy induced insulin resistance^20^ and with lower infant fat accumulation^21^. It is tempting to speculate that the positive effects of exercise during pregnancy on maternal metabolism and fetal development could in part be facilitated through increased upregulation of sOB-R. Interestingly, the greater sOB-R upregulation we saw in women with high activity levels or who exercised more than 150 min was independent of gestational weight gain, which would then agree with studies showing positive outcomes of prenatal exercise without effecting gestational weight gain^46^.

The relationship between physical activity and adiponectin was less clear. In women of normal-weight, a high activity level associated with a larger decrease in adiponectin concentration during pregnancy, which is somewhat surprising. In women with obesity, who started with low adiponectin levels, we saw the opposite relation where exercise was associated with a smaller adiponectin decrease (or even an increase). The few previous studies addressing the relation between maternal exercise and adiponectin levels reported no associations^47–49^.

The strengths of the current study include the longitudinal study design and the thorough characterization of the women at each time point comprising body composition measurements, blood sampling and interviews. Another strength is the use of several types of questionnaires for both general physical activity and also for specific forms of exercise training. There are, however, several limitations to the study. The physical activity and exercise evaluation was based on self-reporting which introduces a risk of reporting bias and also a lack of an objective measure of exercise intensity. Objective measurement methods such as the use of accelerometers and heart rate monitors would be desirable for future projects. There is also a risk for recruitment bias in this type of study. However, the focus of the main study was not on physical activity but on general maternal and child health with a dietary emphasis, which might somewhat limit the bias of recruiting women with a particular interest in exercise. Further, while air plethysmography with gestational-age specific calculations is perhaps the best method available for body composition determination during pregnancy, it does give wide limits of agreement. This needs to be taken into consideration when estimating fat mass changes and looking at its relation with physical activity. Another methodological consideration is the long timeframe of the study. This was controlled for as much as possible with rigorous protocols and consistency in personnel and methods. Finally, the homogeneous study population might be an advantage when looking at differences in a smaller population, but it also means that the generalizability to other ethnicities might be limited.

In conclusion, women with obesity, who are less active than women of normal-weight, may benefit from moderate increases in activity to limit their gestational weight gain. For both groups of women, exercise during pregnancy showed consistent positive association with sOB-R, which may affect pregnancy induced insulin resistance as well as fetal metabolic programming through its regulation of leptin action. Our study overall supports that particularly women with obesity—who are leptin resistant and have a high risk for excessive weight gain, gestational diabetes and increased fetal adiposity—should receive education and practical help in order to increase their physical activity with an emphasis that even moderate increases in physical activity can be beneficial for both maternal and child health.

## Supplementary Information

Supplementary Information.

## Data Availability

The data that support the findings of this study are available from the corresponding author upon reasonable request.

## Abbreviations

IOM: Institute of Medicine
LGA: Large for gestational age
NW: Women of normal weight
OB: Women with obesity
PONCH: Pregnancy Obesity Nutrition and Child Health
SGPALS: Saltin-Grimby physical activity level scale
sOB-R: Soluble leptin receptor
T1–T3: Trimesters 1–3
