# Supplementary material for: Physical activity during pregnancy and association with changes in fat mass and adipokines in women of normal-weight or with obesity
Source: Sci Rep. 2021 Jun 15;11:12549. doi: 10.1038/s41598-021-91980-z (PMC8206069; doi:10.1038/s41598-021-91980-z)
Supplement: Supplementary file 1 — Supplementary Information. [file 41598_2021_91980_MOESM1_ESM.pdf]

Supplemental Material for:

Physical activity during pregnancy and association with changes in fat mass and adipokines in women of normal-weight or with obesity

Ulrika Andersson-Hall[1], Hanna de Maré[1], Freja Askeli[1], Mats Börjesson[2, 3, 4], Agneta Holmäng[1]

[1]Institute of Neuroscience and Physiology, Department of Physiology, Sahlgrenska Academy, University of Gothenburg, 405 30 Gothenburg, Sweden

[2] Centre for Health and Performance, Department of Food and Nutrition, and Sport Science, University of Gothenburg, Gothenburg, Sweden.

[3]Department of Acute and Molecular Medicine, Institute of Medicine, Sahlgrenska Academy, University of Gothenburg, Gothenburg, Sweden

[4]Dept of MGA, Sahlgrenska University Hospital, Region of Västra Götaland, Gothenburg, Sweden

Corresponding author: Ulrika Andersson Hall, Institute of Neuroscience and Physiology, Box 432, Sahlgrenska Academy, University of Gothenburg, 405 30 Gothenburg, Sweden.

E-mail: ulrika.andersson.hall@gu.se

Table S1. Failure analysis of women not included for sOB-R analysis.

	NW women			OB women		
	Included in analysis	Not included in analysis	*p*	Included in analysis	Not included in analysis	*p*
T1 values						
BMI (kg/m^2)	22.2 ± 1.5	21.9 ± 1.4	0.26	34.4 ± 4.0	34.4 ± 3.8	0.94
Fat Mass (kg)	17.0 ± 4.3	16.2 ± 3.9	0.30	46.2 ± 10.8	47.4 ± 10.0	0.71
Leptin (ng/ml)	13.6 ± 6.9	11.6 ± 5.4	0.12	49.0 ± 16.3	55.9 ± 22.2	0.33
Adiponectin (ug/ml)	18.3 ± 7.6	16.1 ± 6.8	0.14	11.3 ± 5.4	10.6 ± 6.3	0.77
T1 to T3 change						
Body Weight (kg)	11.6 ± 3.1	11.1 ± 2.4	0.30	7.8 ± 4.8	7.8 ± 3.4	0.99
Fat Mass (kg)	4.8 ± 3.1	4.3 ± 2.3	0.36	1.2 ± 3.8	0.9 ± 3.8	0.83
Leptin (ng/ml)	7.4 ± 11.6	6.3 ± 9.5	0.61	7.6 ± 22.9	3.3 ± 13.4	0.48
Adiponectin (ug/ml)	-4.5 ± 6.1	-1.9 ± 10.2	0.09	-1.4 ± 3.9	0.7 ± 5.4	0.21

Significance determined with unadjusted t-test. NW, women of normal weight; OB, women with obesity

Table S2. Spearman correlation between metabolic parameters at the start of pregnancy with training reported for the prior 12 months.

	Activity level Leisure		Activity level Work		Walk/Low Impact training (n)		Cardiovascular training (n)		Strength training (n)	
	Rho	*p*	Rho	*p*	Rho	*p*	Rho	*p*	Rho	*p*
NW										
BMI (kg/m^2)	0.022	0.799	-0.005	0.958	0.058	0.509	0.064	0.467	0.044	0.615
Fat Mass (kg)	-0.104	0.233	-0.001	0.990	0.095	0.278	-0.047	0.589	-0.021	0.811
Leptin (ng/ml)	-0.083	0.378	0.156	0.097	0.147	0.120	-0.175	0.063	-0.092	0.335
sOB-R (ng/ml)	0.194	0.078	-0.005	0.964	0.101	0.369	0.013	0.906	0.069	0.540
Adiponectin (ug/ml)	0.025	0.787	-0.058	0.539	0.069	0.470	0.052	0.579	-0.071	0.453
OB										
BMI (kg/m^2)	0.142	0.324	-0.061	0.673	-0.066	0.648	-0.230	0.108	0.077	0.594
Fat Mass (kg)	0.040	0.785	-0.060	0.682	-0.164	0.259	-0.137	0.349	-0.033	0.822
Leptin (ng/ml)	-0.234	0.140	0.183	0.252	-0.004	0.982	-0.182	0.255	-0.209	0.189
sOB-R (ng/ml)	0.277	0.118	0.034	0.851	-0.067	0.712	**0.379**	**0.030**	**0.373**	**0.032**
Adiponectin (ug/ml)	0.145	0.367	0.228	0.152	-0.004	0.979	-0.011	0.944	0.041	0.800

Spearman correlation between self-reported physical activity data and metabolic measurements. NW, women of normal weight; OB, women with obesity.

Table S3. Change in weight, fat mass and adipokines from first to last trimester depending on whether the women reported any *walk or low impact exercise* at their visits in trimesters 2 and 3.

	Training reported in trimester 2				Training reported in trimester 3			
	Yes	No	*p*	p^{adj}	Yes	No	*p*	p^{adj}
NW	*n=64*	*n=70*			*n=66*	*n=69*		
Body Weight	11.4 ± 3.3	11.4 ± 2.5	0.91	0.58	11.6 ± 3.4	11.2 ± 2.4	0.49	0.61
Fat Mass	4.6 ± 3.0	4.6 ± 2.7	0.91	0.54	4.5 ± 3.1	4.7 ± 2.5	0.74	0.53
Leptin	6.0 ± 12.4	8.0 ± 9.8	0.36	0.11	6.8 ± 12.0	7.5 ± 10.3	0.75	0.33
sOB-R*	13.9 ± 14.5	13.8 ± 16.3	0.99	0.99	12.4 ± 14.4	15.5 ± 16.5	0.37	0.49
Adiponectin	-4.4 ± 7.2	-3.3 ± 8.1	0.44	0.68	-4.9 ± 4.8	-2.7 ± 9.4	0.12	0.22
OB	*n=18*	*n=32*			*n=20*	*n=30*		
Body Weight	6.7 ± 4.1	8.5 ± 4.4	0.18	0.12	7.2 ± 4.2	8.2 ± 4.4	0.42	0.27
Fat Mass	0.5 ± 3.3	1.5 ± 4.0	0.36	0.67	0.8 ± 3.5	1.3 ± 3.9	0.70	0.77
Leptin	0.7 ± 13.3	5.9 ± 16.4	0.31	0.43	3.9 ± 17.0	4.3 ± 14.8	0.94	0.56
sOB-R*	10.8 ± 8.6	8.9 ± 5.0	0.51	0.82	12.0 ± 8.7	8.2 ± 4.4	0.18	0.23
Adiponectin	-0.9 ± 4.6	-1.1 ± 4.2	0.88	0.74	-0.1 ± 4.1	-1.5 ± 4.3	0.30	0.44

Significance determined with unadjusted t-test (p) or ANCOVA adjusted for age, BMI in trimester 1, parity and education (p^{adj}). Δ denotes the gestational change from trimester 1 to trimester 3. *sOB-R analysis was made on a subset of women; n= 84 for NW (n=42/42 for yes/no in trimester 2 and n=43/40 in trimester 3) and n=33 for OB (n=12/21 for yes/no in trimester 2 and n=12/21 in trimester 3). NW, women of normal weight; OB, women with obesity

Table S4. Change in weight, fat mass and adipokines from first to last trimester depending on whether the women reported any *aerobic exercise* at their visits in trimesters 2 and 3.

	Training reported in trimester 2				Training reported in trimester 3			
	Yes	No	*p*	p^{adj}	Yes	No	*p*	p^{adj}
NW	*n=44*	*n=90*			*n=39*	*n=96*		
Body Weight	10.9 ± 2.8	11.7 ± 2.9	0.14	0.22	11.2 ± 3.1	11.5 ± 2.8	0.64	0.18
Fat Mass	4.2 ± 3.1	4.8 ± 2.7	0.24	0.27	4.7 ± 2.8	4.5 ± 2.8	0.70	0.81
Leptin	4.6 ± 7.7	8.3 ± 12.1	0.05	0.14	6.5 ± 12.0	7.5 ± 10.6	0.67	0.21
sOB-R	**18.7 ± 18.3**	**11.3 ± 13.0**	**0.03**	**0.03**	15.1 ± 16.6	13.1 ± 14.8	0.57	0.34
Adiponectin	-5.0 ± 4.5	-3.2 ± 8.8	0.23	0.36	-4.0 ± 5.1	-3.6 ± 8.7	0.78	0.81
OB	*n=11*	*n=39*			*n=10*	*n=40*		
Body Weight	7.3 ± 5.2	8.0 ± 4.1	0.65	0.83	6.7 ± 4.4	8.1 ± 4.3	0.37	0.38
Fat Mass	1.4 ± 4.4	1.0 ± 3.6	0.76	0.81	-0.4 ± 3.2	1.5 ± 3.8	0.16	0.18
Leptin	6.6 ± 12.3	3.4 ± 16.3	0.60	0.61	4.4 ± 9.5	4.1 ± 16.9	0.96	0.61
sOB-R	**15.2 ± 7.0**	**7.8 ± 5.3**	**0.003**	**0.01**	11.8 ± 8.8	8.8 ± 5.4	0.24	0.18
Adiponectin	**1.7 ± 4.9**	**-1.8 ± 3.8**	**0.03**	**0.02**	-0.2 ± 5.1	-1.2 ± 4.0	0.55	0.37

Significance determined with unadjusted t-test (p) or ANCOVA adjusted for age, BMI in trimester 1, parity and education (p^{adj}). Δ denotes the gestational change from trimester 1 to trimester 3 *sOB-R analysis was made on a subset of women; n= 84 for NW (n=29/55 for yes/no in trimester 2 and n=32/51 in trimester 3) and n=33 for OB (n=8/25 for yes/no in trimester 2 and n=9/24 in trimester 3). NW, women of normal weight; OB, women with obesity

Table S5. Change in weight, fat mass and adipokines from first to last trimester depending on whether the women reported any *resistance exercise* at their visits in trimesters 2 and 3.

	Training reported in trimester 2				Training reported in trimester 3			
	Yes	No	*p*	p^{adj}	Yes	No	*p*	p^{adj}
NW	*n=50*	*n=84*			*n=31*	*n=104*		
Body Weight	11.3 ± 3.1	11.5 ± 2.7	0.77	0.64	**10.5 ± 3.1**	**11.6 ± 2.8**	**0.05**	**0.01**
Fat Mass	4.5 ± 3.1	4.7 ± 2.7	0.68	0.73	**3.6 ± 3.3**	**4.9 ± 2.6**	**0.03**	**0.02**
Leptin	7.3 ± 9.4	7.0 ± 12.0	0.88	0.40	5.3 ± 7.5	7.8 ± 12.0	0.30	0.45
sOB-R	15.3 ± 17.8	12.7 ± 13.2	0.44	0.22	**17.6 ± 18.9**	**12.1 ± 13.3**	**0.13**	**0.02**
Adiponectin	-5.5 ± 5.2	-2.7 ± 8.7	0.06	0.22	-4.8 ± 5.2	-3.4 ± 8.4	0.39	0.99
OB	*n=9*	*n=41*			*n=7*	*n=43*		
Body Weight	9.8 ± 2.7	7.4 ± 4.5	0.14	0.29	8.8 ± 2.9	7.7 ± 4.5	0.52	0.47
Fat Mass	2.1 ± 3.5	0.9 ± 3.8	0.43	0.17	1.7 ± 3.6	1.0 ± 3.8	0.64	0.10
Leptin	11.9 ± 10.5	2.8 ± 15.9	0.18	0.15	3.7 ± 9.2	4.2 ± 16.1	0.95	0.96
sOB-R	13.7 ± 7.5	8.9 ± 6.1	0.13	0.16	16.4 ± 7.6	8.9 ± 6.1	0.05	0.07
Adiponectin	-1.7 ± 1.8	-0.9 ± 4.6	0.66	0.64	-2.4 ± 2.0	-0.9 ± 4.4	0.51	0.60

Significance determined with unadjusted t-test (p) or ANCOVA adjusted for age, BMI in trimester 1, parity and education (p^{adj}). Δ denotes the gestational change from trimester 1 to trimester 3. *sOB-R analysis was made on a subset of women; n= 84 for NW (n=37/47 for yes/no in trimester 2 and n=27/56 in trimester 3) and n=33 for OB (n=5/28 for yes/no in trimester 2 and n=3/30 in trimester 3). NW, women of normal weight; OB, women with obesity
